# Efficacy of coronary imaging on bifurcation intervention

**DOI:** 10.1007/s12928-020-00701-2

**Published:** 2020-09-07

**Authors:** Kensuke Takagi, Ryoji Nagoshi, Byeong-Keuk Kim, Woong Kim, Yoshihisa Kinoshita, Junya Shite, Yutaka Hikichi, Young Bin Song, Chang-Wook Nam, Bon-Kwon Koo, Soo-Joong Kim, Yoshinobu Murasato

**Affiliations:** 1 Department of Cardiology, Ogaki Municipal Hospital, Ogaki, Japan; 2 Department of Cardiology, Osaka Saiseikai Nakatsu Hospital, Osaka, Japan; 3 Department of Cardiology, Yonsei Severance Hospital, Seoul, South Korea; 4 Department of Cardiology, Yeungnam Medical Center, Daegu, South Korea; 5 Department of Cardiovascular Medicine, Toyohashi Heart Center, Toyohashi, Japan; 6 Department of Cardiology, Saga University, Saga, Japan; 7 Department of Cardiology, Samsung Medical Center, Seoul, South Korea; 8 Division of Cardiology, Department of Internal Medicine, Keimyung University Dongsan Hospital, Daegu, South Korea; 9 Department of Internal Medicine and Cardiovascular Center, Seoul National University Hospital, Seoul, South Korea; 10 Division of Cardiology, Department of Internal Medicine, Kyung Hee University College of Medicine, Kyung Hee University Hospital, 23, Kyungheedae-ro, Dongdaemun-gu, Seoul, 02447 South Korea; 11 Department of Cardiology and Clinical Research Center, National Hospital Organization, Kyushu Medical Center, 1-8-1, Jigyohama, Chuo, Fukuoka 810-8563 Japan

**Keywords:** Intravascular ultrasound, Optical coherence tomography, Coronary bifurcation, Percutaneous coronary intervention

## Abstract

During the coronary bifurcation intervention procedure, imaging including intravascular ultrasound and optical coherence tomography is essential to provide precise anatomy of the lesion and morphological information. This consensus document between the Korean Bifurcation Club and the Japanese Bifurcation Club summarizes practical guidelines and current evidences on lesion assessment, device selection, procedural guidance, and the optimization of bifurcation intervention by the imaging.

## Introduction

Percutaneous coronary intervention (PCI) for bifurcation lesions remains challenging because of a relatively low success rate, high incidence of procedural complications and inferior clinical outcomes compared to those in non-bifurcation lesions; even in the new-generation drug-eluting stent (DES) era [1]. In bifurcation lesions, coronary angiography cannot accurately visualize the carina area due to overlapping of the main vessel (MV) and side branch (SB), which might limit the accurate assessment of atherosclerotic involvement. On the contrary, intravascular imaging including intravascular ultrasound (IVUS) or optical coherence tomography (OCT) is useful in guiding the PCI strategy by offering helpful pre-procedural information such as lumen and vessel dimensions, and lesion characteristics during PCI. Furthermore, Imaging-guided PCI could provide more favorable outcomes than angio-guided PCI by allowing optimal expansion and apposition of the stent as well as its appropriate landing zone. In this first consensus document between the Korean Bifurcation Club (KBC) and the Japanese Bifurcation Club (JBC), we include a scientific discussion, practical guidelines, and current evidences on debatable issues about IVUS and OCT imaging assessment of bifurcation lesions.

## Impact of imaging-guidance on bifurcation intervention

There are several mechanisms responsible for treatment failure in bifurcation lesions. Stent under-expansion due to insufficient preparation of severely calcified or hard fibrous plaques in lesions is one of the most leading causes for in-stent restenosis (ISR). Edge dissection after oversized stenting or aggressive post-dilatation also increases the risk of ISR. Coronary angiography is not able to clearly elucidate these contributing factors for stent failure and frequently shows ambiguity during bifurcation stenting [2]. In addition, it has limitations in assessing the SB ostium, overlapping stent segments, lesion coverage, stent apposition, and wire position. However, intravascular imaging studies could provide essential information for the lesion characteristics and the dimensions of the vessel and lumen in bifurcation lesions. In addition, they could help the assessment of anatomic configuration, selection of treatment strategy, and evaluation of post-treatment results including stent expansion and apposition, which are very important in the treatment of bifurcation lesions [3, 4]. Indeed, several meta-analyses of randomized trials and observational registries showed the superiority of imaging-guided PCI in complex procedures, including left main (LM) stem and bifurcations, compared to angiography-guided PCI. According to the data from a Korean multicenter bifurcation registry, IVUS-guided PCI with DES significantly reduced the incidence of death or MI compared with angiography-guided PCI in a cohort of bifurcation lesions [5]. Basically, both pre- and post-procedural observations in MV and SB are recommended with the exception of post-procedural observation of the SB jailed by the MV stent due to the risk of distortion or fracture of the stent. In the pre-procedural observation, the following assessments facilitate optimal selection of devices and PCI strategy: (1) measurement of dimensions for lumen and vessel in MV and SB; (2) assessment of atherosclerotic plaque morphology, burden, longitudinal distribution, and negative remodeling; (3) detection of angiographically silent disease; and (4) the risk of SB compromise. In the post-procedural observation, the following assessments are required to optimize the procedure: (1) stent apposition, (2) stent expansion, (3) full lesion coverage by the stent, (4) stent edge dissection, (5) plaque prolapse inside stent, (6) SB residual stenosis and dissection, and (7) optimal guidewire (GW) recrossing before SB dilation and subsequent adequate clearance of jailing struts after SB dilation.

## Advantage of IVUS

In the current guidelines, IVUS-guided PCI is recommended for complex lesions including LM, bifurcation, long lesions, and chronic total occlusions due to particular challenges in angiographic evaluation and procedural complexity [6]. The ‘Impact of Intravascular Ultrasound Guidance on Outcomes of Xience Prime Stents in Long Lesions (IVUS-XPL)’ trial showed the superiority of IVUS-guided PCI in 1400 patients with long coronary lesions compared with angiography-guided PCI [7]. Furthermore, the ULTIMATE Trial, which randomly assigned 1448 patients to IVUS usage, also showed that IVUS-guided DES implantation significantly improved clinical outcomes of all-comers compared with angiography guidance [8]. On meta-regression analysis, IVUS lowered the mortality rate in patients with complex lesions or acute coronary syndrome [9].

On the other hand, the usefulness of IVUS is emphasized in LM PCI, especially when complex PCI is necessary. Extensive evidence supports the use of IVUS-guided LM PCI in non-randomized studies. Park et al. demonstrated that IVUS-guided PCI tended to induce lower mortality rates without demonstrating a difference in myocardial infarction or target lesion revascularization (TLR) [10]. De La Torre Hernandez et al. clarified that the use of IVUS in PCI for complex LM lesions, from the data of propensity-score matched population, could significantly reduce the risk of stent thrombosis (ST) [11]. Most recently, Andell et al. reported IVUS-guidance reduced the incidence of a combined primary endpoint of mortality, ST, and restenosis in the LM PCI registry over a period of 5 years [12]. Therefore, current guidelines recommend that IVUS-guided PCI is necessary for LM bifurcation lesions to reduce adverse events. Table 1 summarizes the usefulness of IVUS during PCI for LM and bifurcation lesions.

**Table 1:** Characteristics of lesions, and the procedure and clinical outcome in each publication concerning the comparison between Angio-guide and IVUS-guide in coronary bifurcation PCI

	Year of publication	Lesion subset	Number of patients	Distal Bifurcation (%)	Study type	ACS (%)	Follow-up	Clinical outcome IVUS- vs. Angio-guide
Park SJ et al.[10]	2009	LM	145/145	53.0	Propensity score matched	61.2	3 years	Death 4.7% vs.16.0%
De La Torre Hernandez JM, et al. [11]	2014	LM	505/505	44.2	Propensity score matched	60.0	3 years	Cardiac death/MI/TLR 11% vs. 19%
Gao XF, et al.[78]	2014	LM	291/291	86.4	Propensity score matched	NA	1 year	Cardiac death/MI/TLR 16.2% vs. 24.4%
Tan Q, et al.[79]	2015	LM	61/62	53.7	RCT	68.3	2 years	Cardiac death/MI/TLR 13.1% vs. 29.3%
Kim SH, et al. [80]	2010	Non-LM bifurcation	758/758	NA	Propensity score matched	52.0	4 years	All cause of mortality HR 0.31 (95% CI 0.13–0.74), Very late stent thrombosis 0.4% vs 2.8%
Kim JS et al. [5]	2011	Non-LM bifurcation	487/487	NA	Propensity score matched	53.2	23.7 months	Death or MI 3.8% vs 7.8% HR 0.44, 95% CI 0.12–0.96,
Chen SL et al. [81]	2013	2-stent	123/123	NA	Propensity score matched	87.3	1 year	Stent thrombosis 0% vs 4.9% ST-elevation MI 2.4% vs. 9.8%

LM = left main, RCT = randomized control trial, MI = Myocardial Infarction. TLR = Target Lesion Revascularization, NA = not available

A few studies that used greyscale or virtual histology IVUS demonstrated that plaque accumulation and vulnerability were more frequently shown in the proximal MV than in the distal MV in a bifurcation lesion [13, 14], suggesting that plaque rupture is more likely to occur in the proximal MV than in the other segments. Indeed, the CROSS trial revealed that angiographic ISR was frequently observed at the proximal stent edge after PCI with a single stent strategy in a bifurcation lesion [15]. IVUS could provide valuable information for plaque vulnerability and burdens in the bifurcation before stenting.

## Advantage of OCT

Since the introduction of the frequency-domain type, OCT has been used more frequently in the guidance of PCI due to generation of high-resolution images with high speed pull back and convenient usability. In the latest guidelines of the European Society of Cardiology and European Association for Cardio-Thoracic Surgery, OCT as well as IVUS are recommended for procedural optimization as class IIa [16]. For OCT image acquisition, vessel flushing is necessary to remove blood and low-molecular-weight dextran is available as subsidies with contrast medium for frequent observations required in the bifurcation PCI [17, 18]. The OCT can provide a clear visualization of coronary plaques and accurate measurement of reference lumen diameter and lesion length [19, 20]. In the clinical study OPUS-CLASS, IVUS presented a larger minimal lumen diameter and area than OCT by 9% and 10%, respectively [19]. In comparing between OCT and IVUS measurements in the phantom model, OCT was equal to the actual lumen area of the model, while IVUS overestimated and was less reproducible [19]. Hence, these characteristics are useful to decide stent size, length, landing zone, balloon size and necessity of distal protection [21]. For example, OCT can characterize the plaque components such as lipid rich or calcified plaque more clearly and we can avoid the area which contains vulnerable plaques for stent landing zone [22]. In addition, a current angio-coregistration system which reflects the location of the OCT camera on the coronary angiogram is helpful to realize stent landing zone and minimize geographic miss. After MV stenting, OCT is useful for the assessment of stent expansion, apposition, in-stent tissue protrusion, vessel dissection, GW recrossing position, and stent deformation. For the effective proximal optimization technique (POT) that facilitates optimal GW recrossing with the wide opening of the jailed struts in the SB ostium, OCT can provide useful information concerning balloon size, the length between carina and stent proximal edge, and accurate position of the balloon distal marker on the carina [23, 24]. Previous bench tests revealed that GW recrossing to the distal cell adjacent to the carina, led to wide SB opening with less malapposition after kissing balloon inflation (KBI); and GW recrossing to more proximal cell introduced protrusion of the jailed struts into the MV [24]. Clear visualization of the GW and stent strut in the three dimensional (3D) OCT image has a great impact on an accurate assessment of optimal GW recrossing which reduces stent malapposition significantly (9.5% vs 42.3% in the angiography-guided group, *p* < 0.0001) [25]. Initial angiography-guidance failed in GW recrossing to the optimal cell in 33–35% while OCT guidance improved the success rate up to 90–100% [26–28]. In the 3D-OCT bifurcation registry, 3D-OCT guidance allowed significantly better attainment of optimal distal GW recrossing without increasing contrast dye volume and operation time, compared with conventional 2D-OCT guidance [27, 28].

## Comparison of OCT vs. IVUS

There are a few studies comparing OCT and IVUS as guides for PCI (Table 2) [29–31]. The OPINION study, which included bifurcation lesions in 38% of cases, revealed no statistical difference in target vessel failure between IVUS and OCT guidance [30]. Since OCT provides higher resolution images, it is useful to detect stent failure and to optimize the procedure. Unfortunately, the drawbacks of OCT were limited penetrating depth and necessity of blood clearance for the image. Hence, LM bifurcation lesion has been considered unsuitable for the OCT guidance due to more artifacts included, however, the OCT image quality in LM bifurcation was not inferior to those in non-LM bifurcation in recent studies [27, 28]. 3D- OCT guidance provided significantly less incomplete strut apposition than 2D- OCT guidance, after LM bifurcation stenting, followed by KBI with the assessment of GW recrossing point (18.7 ± 12.8% vs 10.3 ± 8.9%; *P* = 0.014); which was not statistically significant in whole bifurcation cases (14.5 ± 13.6% vs 10.0 ± 9.0%; *P* = 0.077) [28]. Another advantage of OCT guidance is a clear visualization of the calcium border and accurate measurement of calcium thickness that are useful for adequate lesion preparation using a rotablator in calcified lesions [32]. Calcified plaques with < 0.67 mm thickness were able to be dilated with cracks by balloon inflation [32]. Generally, IVUS guidance is more suitable for larger vessels and aorta-ostial lesions due to limitations of complete vessel flushing or adequate assessment in OCT guidance.

**Table 2:** Clinical studies comparing OCT- and IVUS-guided PCI without excluding bifurcation lesions

Study name Published year	Study design	Number of Pts	Bifurcation (%)	Primary Endpoint	Results
ILUMIEN II 2015 [31]	Post hoc matched-paired analysis of ILUMIEN I and ADAPT-DES	Matched groupsOCT: 286 IVUS: 286	OCT: 97 (33.9) IVUS: 93 (32.5)	Stent expansion (%) (MSA / mean lumen area)	OCT: 72.8% [63.3–81.3] IVUS: 70.6% [62.3–78.8] (median [IQR], *p* = 0.29)
ILUMIEN III 2016 [29]	Randomized, controlled multi-center study	OCT: 158 IVUS: 146 Angiography: 146	Unknown	MSA measured by OCT	OCT: 5.79 [4.54–7.34) mm^2^ IVUS: 5.89 [4.67–7.80) mm^2^ Angiography 5.49 [4.39–6.59] mm^2^ OCT vs. IVUS: non-inferior (*p* = 0.001), but not superior (*p* = 0.42)
OPINION 2017 [30]	Randomized, controlled multi-center study	OFDI: 414 IVUS: 415	OFDI: 154 (37.4) IVUS: 157 (38.8)	TVF (Composite of cardiac death, target-vessel related myocardial infarction and ischemia-driven target vessel revascularization)	OFDI: 21 (5.2%) IVUS: 19 (4.9%) OFDI vs. IVUS: non-inferior (*P* = 0.042)

MSA: Minimum stent area, IQR: interquartile range, TVF: Target vessel failure

## Role of IVUS and OCT as predictors of TLR/restenosis

**Fig. 1:**
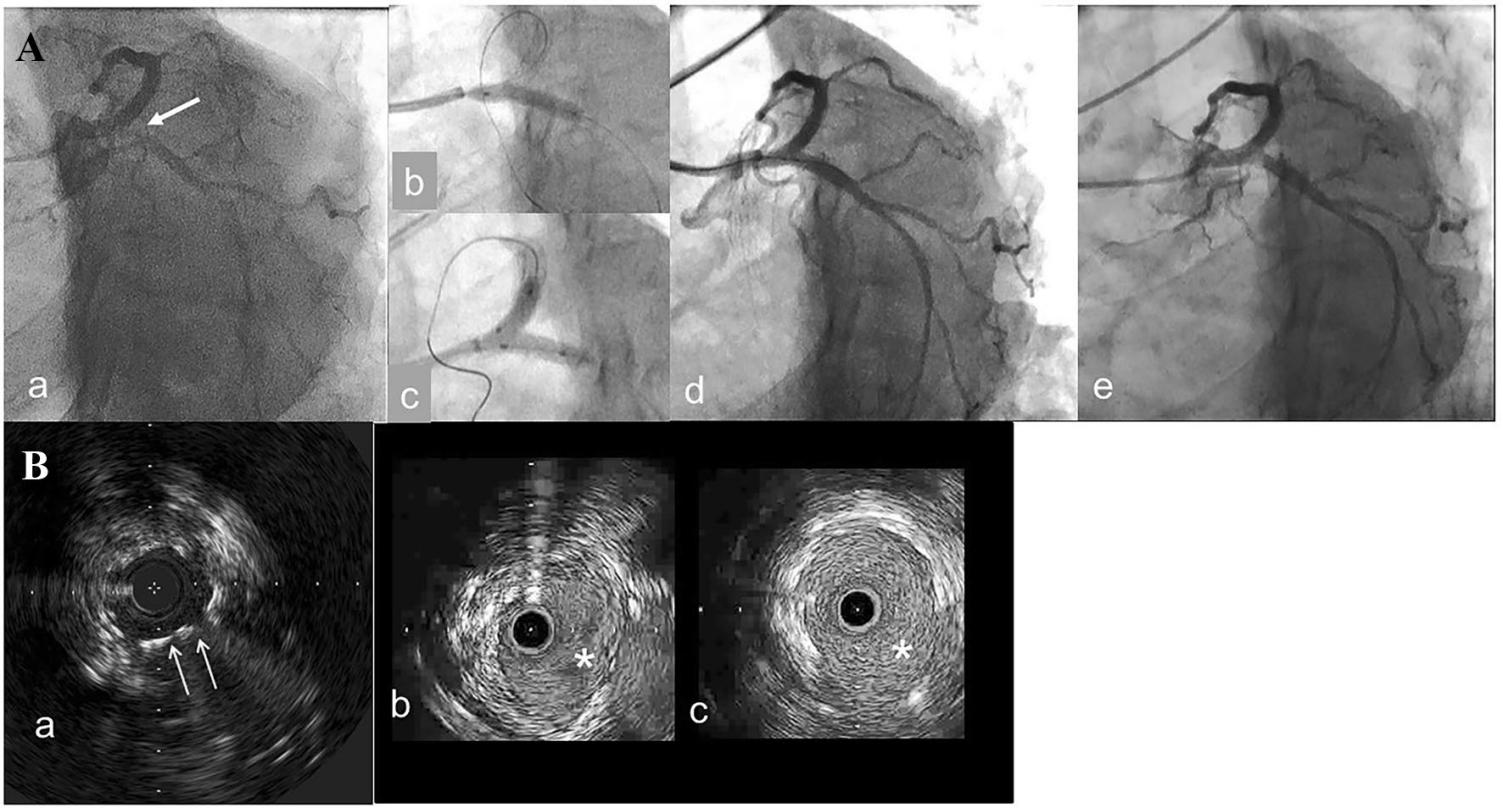
A representative case of IVUS-guided treatment for the in-stent restenosis (ISR) after modified T-stenting in left main (LM) bifurcation performed 9 months before. Coronary angiography (CAG) showed tight ISR (A-a) and IVUS showed stent under expansion with minimal stent area (MSA) of 3.6mm2 in left circumflex (LCX) ostium as well as remained metallic carina (B-a, arrows). After implantation of a 3.5/18 mm zotarolimus-eluting stent crossing over left anterior descending artery (LAD) (A-b), kissing balloon inflation using two 3.5 mm non-compliant balloons was performed at 16 atm (A-c), which resulted in no angiographic stenosis (A-d). In the IVUS observation, both LAD (B-b) and LCX (B-c) were well expanded with MSA of 8.2mm2 and 10.5mm2, respectively, which led to a marked reduction of the metallic carina (asterisks). CAG at 1-year follow-up demonstrated no significant restenosis in the treated site

In general, stent under-expansion is established as a major predictor of stent failure [33, 34]. A threshold of absolute minimum stent cross-sectional area (MSA) in IVUS analysis can be used to prevent target vessel failure involving TLR and ST. The cut-off value for MSA is 4.0–5.7 mm^2^ in PCI using first-generation DES [35–37]. Focusing on LM lesions, Kang SJ et al. reported that the MSA cutoffs used to predict restenosis on a segmental basis were 5.0 mm^2^ (ostial left circumflex artery, LCx), 6.3 mm^2^ (ostial left anterior descending artery, LAD), 7.2 mm^2^ (distal LM), and 8.2 mm^2^ (proximal LM) in IVUS analysis [38]. As shown in Fig. 1, stent under-expansion in LCX ostium and remaining metallic carina after 2-stenting in LM is likely to generate restenosis and IVUS-guided stent expansion was therefore useful. In previous randomized trials, IVUS criteria for optimal stent expansion were defined as a MSA greater than or equal to the distal reference, that is, a MSA > 80 to 90% of the average reference. Importantly, approximately one-third of patients did not achieve the predefined criteria of stent expansion in selected randomized trials of imaging-guided PCI [6]. In the IVUS-XPL trial, optimization was achieved in half of the lesions, which showed better clinical outcomes than those in which the criteria were not achieved [7]. Edge restenosis was predicted by residual plaque burden of > 51.6% to 54.5% in the DES-stented segment [39, 40]. Therefore, the benefits of IVUS-guided PCI are gaining sufficient MSA and accurate setting of stenting zone.

The automatic measuring function in OCT is helpful to detect stent under-expansion immediately, which is established as a major predictor of stent failure [6]. Tapering from proximal to distal MV should be considered in assessing stent expansion in the bifurcation lesion. However, most of the imaging studies focused on the association between MSA and clinical events [34–38, 41]. The current version of OCT equips novel volumetric stent expansion analysis- referring to vessel tapering based on the H–K equation (Dmo^7/3^ = Dmb^7/3^ + Dsb^7/3^, where Dmo, Dmb and Dsb were the diameter of mother vessel, main branch and SB, respectively) [42, 43]. With this method, the expansion index is calculated as the ratio of actual lumen area to the ideal lumen, and the minimum expansion index (MEI), demonstrated a strong correlation with device-oriented cardiac events with a cut-off value of < 73.3% [43]. This novel tapered vessel algorithm is applicable for device selection and optimization according to the vascular branching law in bifurcation stenting.

In bifurcation lesions, the predictors of TLR are more complicated because there are numerous complex bifurcation techniques and the presence and severity of SB leads to more complicated interpretations. Three mechanisms for restenosis were noted. First, ISR was caused by stent under-expansion due to insufficient preparation of severely calcified or hard fibrous plaque lesions. POT using an appropriate balloon size by intravascular imaging could more optimally and symmetrically dilate stent struts and prevent low shear stress generation, thus resulting in decreased ISR [38, 44, 45]. Second, edge dissection after oversized stenting or aggressive post-dilatation increases the risk of restenosis. Intravascular imaging-guidance could offer accurate lesion assessment and appropriate selection of device size which can prevent this kind of complication. Third, although the majority of patients with LM lesions had bi-directional plaque extension into the ostial LM and proximal LAD on IVUS observation [46], mild to moderate lesions in the proximal LM are likely to be ignored for stent coverage, resulting in proximal edge restenosis [47]. Therefore, intravascular imaging which can detect hidden, unclear plaque extension in angiography could help decide the stent landing zone. In fact, Takagi et al. showed the efficacy of the combination of POT and full-coverage ostial LM on the reduction of ISR in the ostial LM compared to propensity score-adjusted group that was not treated with this strategy [HR, 0.34 (95% CI, 0.15–0.76), *p* = 0.008] [48].

An additional two-stent strategy is necessary in 3–47% of cases after provisional stenting [49–51]. SB dissection and bail-out two-stent deployment occurred in 10.5% and 5.6% after KBI, respectively, even when dedicated IVUS-guided KBI was performed [52], supporting the usefulness of imaging-guidance to avoid unnecessary bailout two-stenting. Large edge dissections (more than medial layer) detected by IVUS are reportedly associated with early ST [53].

The KBI is crucial in the two-stent strategy. However, in true bifurcation lesions treated with provisional single stenting, the impact of KBI remains controversial according to previous studies [54–56]. Although KBI has certain benefits of stent expansion, modifying carina shift, restoring stent shape, compressing plaque at the ostial SB, and apposing struts to the proximal MV, there are potential concerns regarding the unfavorable impacts of KBI such as vessel dissection, asymmetric expansion, and deterioration of rheological stress due to overdilation and subsequent elliptical deformation [23, 57–59]. In the J-REVERSE trial, the KBI group obtained a greater luminal volume in the proximal MV and demonstrated less binary SB restenosis (9.7% vs. 21.0%, *p* = 0.0004), which was beneficial for both true and non-true bifurcation lesions. They emphasized that accurate assessment of the bifurcated vessel in terms of vessel size, plaque and intima by IVUS lead to optimal KBI treatment without increasing MV events [52]. In addition, KBI reduced proximal-segment luminal narrowing due to homogeneous neointimal distribution and fewer jailed struts at 9-month follow-up [60]. This could be partly explained by optimal balloon size guided by intravascular imaging for KBI.

## Role of IVUS and OCT at predicting SB compromise

Diameter stenosis at the SB ostium and smaller carina angles is associated with SB compromise after MV stenting [61–63]. Furukawa et al. reported that IVUS could identify the presence of plaques truly involved in SB ostium that were not detected by angiography, which was associated with the SB occlusion [64]. In the bench test, Vassilev and Gil demonstrated that carina shift is a major mechanism of SB occlusion after stent crossover and that the diameter of MV at distal site of the junction and percent diameter stenosis of SB ostium are correlated with acute SB occlusion just after stent implantation from angiography [65]. On the other hand, recent IVUS analyses found that the main cause of SB compromise is a mixture of carina shift driven by distal MV lumen expansion and plaque shift [66, 67], which is not completely rectified by KBI. In the IVUS sub-study of the J-REVERSE registry, carina shift was more frequently found in cases with SB ostial residual stenosis after KBI than in those without it (37% vs. 11%) [66]. Independent predictors for SB residual stenosis in the pre-procedural IVUS observation were negative-remodeling at distal MV, plaque -burden at distal MV, and plaque-burden at SB ostium [66]. In addition, another IVUS study demonstrated that SB plaque burden was asymmetric and likely to present at the opposite side of flow-divider with low shear stress and SB negative remodeling was frequently encountered in the complex bifurcation lesion with extended SB lesion [68]. Therefore, IVUS, not angiography, is more helpful in precise decision making for bifurcation PCI strategy.

In cases of LM distal bifurcation, careful IVUS imaging usually showed continuous plaques from the LM into the proximal left anterior descending artery (LAD) was seen in 90% and from the LM into the LCx in 66%, with the disease from the LM into both the LAD and LCx in 62% [46]. Of these LM bifurcations, the patients who have a “vulnerable” carina—the eyebrow sign [69] or significant calcium [70] identified by IVUS longitudinal reconstruction, are at particular risk of adverse carina shifts towards the LCx.

The pre-procedural OCT findings are also helpful to predict SB compromise after cross-over stenting as shown in Table 3. More plaque burden, particularly calcified plaque in the MV opposite to the SB orifice [71], narrower carina tip angle and shorter length between proximal branching point to carina tip are predictors of carina shift [72]. The parallel type in which the proximal course of SB is concealed behind carina in the 3D perpendicular image of the SB is more likely to be associated with carina shift than the perpendicular type in which proximal SB is visualized over the carina [73].

**Table 3:** OCT studies identifying predictors of side branch compromise

Author Published year	Number of lesions / complications	Results (SB compromise vs. non-compromise)	Predictors
Watanabe et al 2014 [72]	52 / 22	Frequent eccentric plaque distribution opposite to SB: 77.3% vs. 16.7% Smaller CT (carina tip) angle: 29.58° vs. 65.08° Shorter BP-CT length (length between proximal branching point to CT): 1.20 mm vs. 2.25 mm	CT angle < 50° BP-CT length < 1.70 mm
Fujino et al 2014 [71]	75 / 31	Smaller bifurcation angle: 48.55 ± 20.26° vs. 65.58 ± 33.98° Greater % DS of SB in pre- and post-PCI More calcified plaque	Bifurcation angle < 70° Pre-PCI, SB % DS Calcified plaque in the MV
Kini et al 2017 [82]	30 / 10	Frequent lipid rich plaques (lipid arc > 90%): 100% vs. 64% Greater maximal lipid arc: 257°vs. 132° Lipid rip-rich plaque located contralateral to the SB: Proximal MV; 50% vs. 5%, Distal MV; 30% vs. 10%	Maximal lipid arc Lipid rich plaque contralateral to SB ostium

SB: Side branch, MV: Main vessel, %DS: % diameter stenosis

## Efficacy of 3D-OCT on optimal bifurcation stenting (Table 4 and Fig. 2)

**Table 4:** Clinical studies on bifurcation PCI under 3D-OCT assessment

Author Published year	Study design	Enrolled cases	Results
Okamura et al 2014 [26]	Retrospective, single-center study	22	Accurate assessment of GW recrossing by 3D-OCT: 18/22 (81.8%) Stent configuration over SB orifice: free carina (FC) type; no link at carina (n = 7) vs link-connecting (LC) type; the existence of link at carina (n = 6) %ISA after KBI: FC 0.7 ± 0.9% vs. LC 12.2 ± 6.5%
Okamura et al 2018 [27]	Prospective, multi-center study	105	Distal cell GW recrossing under OCT guidance: 83% %ISA: distal GW recrossing 6.3 ± 6.0% vs. proximal 17.1 ± 10.1% FC type with distal recrossing (LFD group, n = 54) vs. the other cases (non-LFD group, n = 51) %ISA: 6.7 ± 5.9% vs. 17.0 ± 10.5% SB restenosis at follow-up: 8.3% vs. 20.5%, p = 0.1254
Nagoshi et al 2018 [28]	Retrospective, multi-center study	150	Distal cell GW recrossing: 2D-OCT guidance 75.6% vs. 3D-OCT guidance 91.7% %ISA: 2-D OCT vs. 3-D OCT SB: 14.5 ± 13.6% vs 10.0 ± 9.0% Left main bifurcation: 18.7 ± 12.8% vs 10.3 ± 8.9% Independent contributors to ISA: Link-connecting type, distal GW recrossing, age
Kume et al 2018 [83]	Observational, single-center study	29	FC type (n = 18) vs. LC type (n = 11) SB orifice obstruction by neointima at 18-month follow-up: 9.5 ± 22.1% vs. 26.8 ± 21.9%
Fujimura et al 2018 [84]	Retrospective, single-center study	37	Main vessel stenting followed by KBI, follow-up OCT at 6–12 month LFD vs. non-LFD SB ostial area gain: + 0.43 mm^2^ vs. -0.65 mm^2^, + 9.47% vs. -13.77%

*OCT* optical coherence tomography, *GW* guide wire, *KBI* kissing balloon inflation, *ISA* incomplete stent apposition, *SB* side branch

**Fig. 2:**
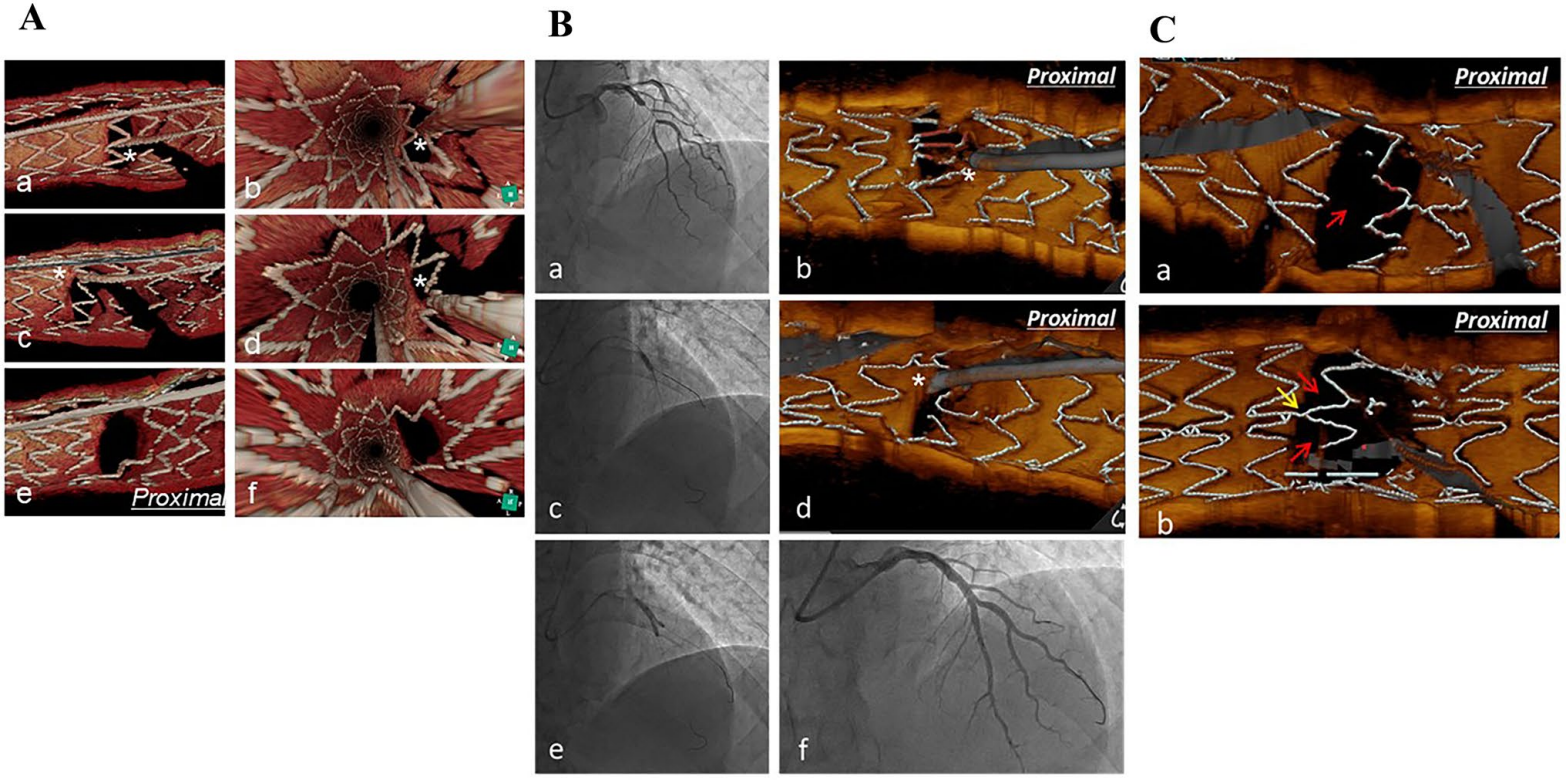
**A** PCI for Medina (1,1,0) lesion in the left anterior descending artery (LAD)—diagonal branch (Dx) bifurcation under OCT guidance. a Pre-procedural angiography. b 3-D OCT image after guidewire (GW) recrossing toward Dx following stent implantation, which demonstrated the proximal cell GW recrossing. c POT with 3.5 × 8 mm balloon. d 3-D OCT image after proximal optimization (POT) and the second GW recrossing toward Dx, which showed the GW recrossing into the distal cell which was enlarged by POT. e Kissing balloon inflation with 2.75 mm and 2.5 mm balloons in LAD and Dx respectively. f Final angiogram. **B** Two patterns of stent configuration over side branch orifice. a Link-free carina type, no link-connection is presented between carina and proximal adjacent struts (red arrow). b Link-connecting carina type, link-connection is located between carina and proximal adjacent strut (yellow arrow), which results that distal SB orifice is divided into 2 spaces by the link (red arrows)

The assessment of stent configuration over SB orifice and GW recrossing position with 3D OCT imaging before KBI provides important information to achieve optimal bifurcation stenting (Fig. 2a). Appropriate POT enlarges the distal site of jailed struts, which increases the likelihood of optimal distal wiring (Fig. 2b) [23]. In the 3D-OCT Registry, 3D-OCT guidance allowed significantly better attainment of optimal distal GW recrossing without an increase of contrast dye volume and operation time compared with the conventional 2D-OCT guidance despite of more performance of GW recrossing [27, 28]. Stent configuration was classified into two patterns. One is link-free carina type, which has no link connection on the carina, and the other is link-connecting carina type, in which the link connection is located between the carina and proximal stent strut (Fig. 2c) [27, 28, 74]. Distal GW recrossing led to better stent apposition to the lateral wall after KBI in the Link-free carina type, while in the Link-connecting type, there was no difference in stent apposition regardless of GW recrossing position [27, 28]. In addition, KBI with distal GW recrossing in the Link-connecting type has a potential risk of stent deformation [75]. Efficacy of KBI after cross-over stenting has been controversial because some randomized studies indicated neutral or adverse effects of KBI on clinical outcome compared to non-KBI procedures, which left jailed struts over the SB orifice [15, 56]. However, the fact that the stent struts at ostial LCx after LM cross-over stenting impacted the narrowing of the ostial area at follow-up OCT study [76], and main pathological predictors for LM stent failure are malapposition and struts crossing an ostial LCx [77] supported the importance of the reduction of stent struts jailing the SB. The 3D- OCT imaging facilitates the achievement of complete removal of jailed struts and fully apposed struts in the bifurcation segment, which may lead to improvement of clinical outcome of the KBI compared to 2D imaging or angiography guidance. Such perspective is more important in two-stent strategy, which requires KBI with high pressure. The 3D-OCT guidance is also effective for optimal GW recrossing both after the first and second stent deployment in the two-stenting technique, which has the potential of improving the clinical outcome at long-term follow-up. The 3D-OCT guided PCI by non-expertise operators still has the following limitations: 1) Less identification of internal elastic lamina in severely diseased vessels is likely to lead to smaller device selection compared to IVUS [30], 2) More usage of contrast medium for vessel flushing has a possible risk of worsening renal function, and 3) Incomplete blood flushing in large vessels or shadow of the GW in 3D images is prone to inaccurate assessment of stent malapposition or GW recrossing position.

## Recommended procedure

i. **IVUS-guided bifurcation PCI.**

**Fig. 3:**
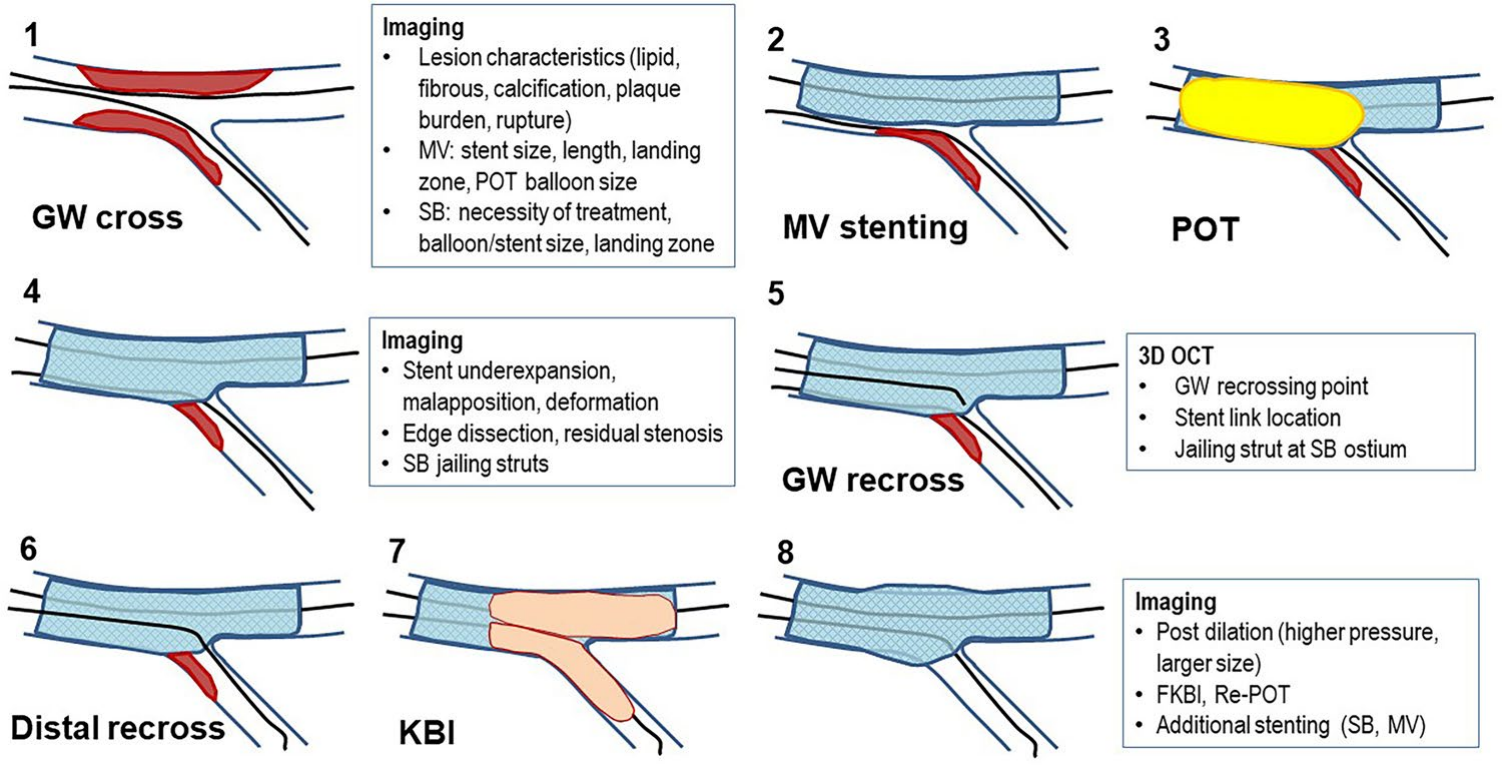
Flow of imaging-guided provisional bifurcation stenting. GW: guide wire, MV: main vessel, POT: proximal optimization technique, SB: side branch, FKBI: final kissing balloon inflation

**Fig. 4:**
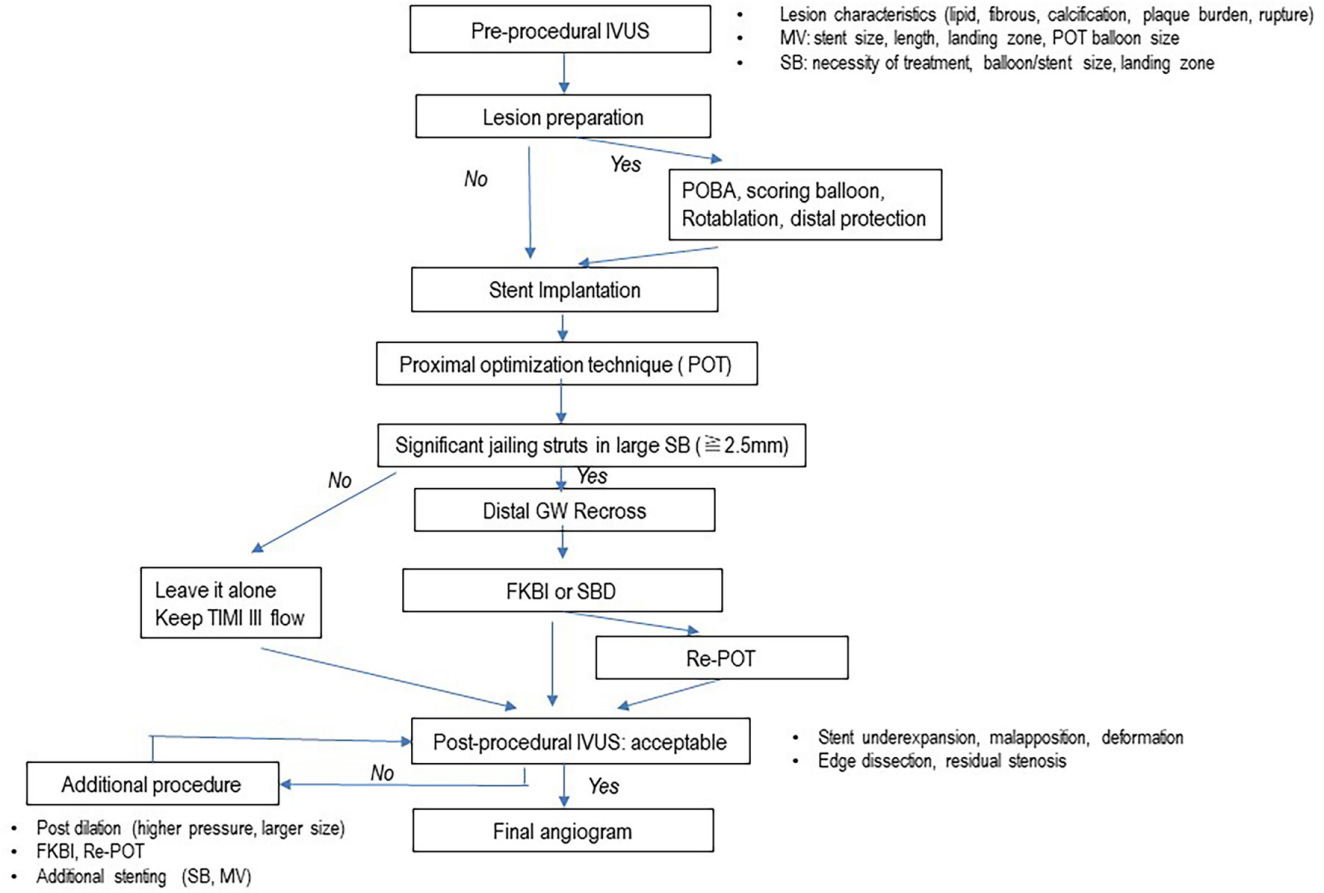
Flow of IVUS-guided bifurcation PCI

As shown in Figs. 3 and 4, pre-procedural observation in both MV and SB is recommended for accurate assessment of lesion morphology, lumen dimension, device selection, as well as a landing zone and decision for SB treatment. If lesion preparation is necessary, pre-dilation by non-compliant or scoring balloon, rotablation or distal protection should be considered. Stent implantation according to the distal MV reference is followed by the POT with a short balloon dedicated by the IVUS. In cases of significant jailing struts in the SB ostium with a size of ≧2.5 mm, distal GW recrossing is attempted. Final KBI or simple SB dilation follows optimal GW recrossing and POT is performed as correction of stent deformation. When post-procedural failure is found in IVUS, such as stent under-expansion, malapposition, deformation, and edge dissection or residual stenosis, optimization of the procedure should be added and reassessed by the IVUS after the procedure.

ii. **OCT-guided bifurcation PCI**

**Fig. 5:**
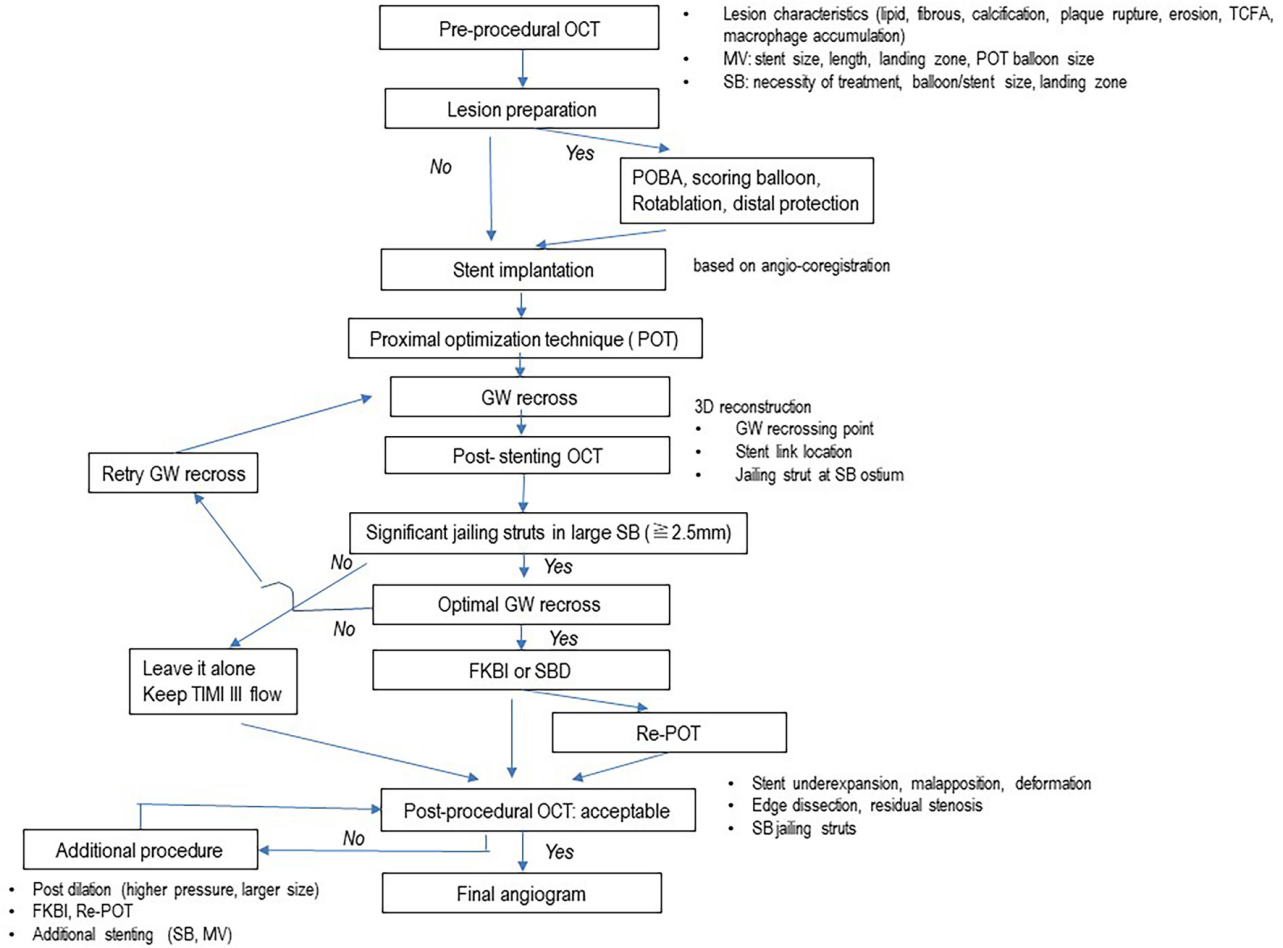
Flow of OCT-guided bifurcation PCI

As shown in Figs. 3 and 5, the recommended OCT-guided bifurcation PCI is basically similar to IVUS-guided PCI, whereas the assessment of GW recrossing point, link-connection and jailing struts on the SB ostium using 3D images is added as a more meticulous step. When suboptimal GW recrossing and SB ostial dilation or stent malapposition/deformation is found, additional optimization procedures should be considered.

## Summary

The accurate morphological assessment of MV and SB using an intra-luminal imaging device before and after the procedure are important for optimization of the device during complex bifurcation PCI procedures. Imaging guidance can provide adequate stent expansion, less malapposition, appropriate stent landing, and treatment of dissection; which lead to improvement of clinical outcome. Moreover, the assessment of the recrossing position and stent link location with 3D- OCT imaging has the potential to improve the clinical outcome due to optimal SB treatment.
